# Skin Abscess due to *Serratia marcescens* in an Immunocompetent Patient after Receiving a Tattoo

**DOI:** 10.1155/2015/626917

**Published:** 2015-08-18

**Authors:** J. Diranzo García, J. Villodre Jiménez, V. Zarzuela Sánchez, L. Castillo Ruiperez, A. Bru Pomer

**Affiliations:** Servicio de Cirugía Ortopédica y Traumatología, Consorcio Hospital General Universitario de Valencia, Avenida Tres Cruces 2, 46014 Valencia, Spain

## Abstract

The incidence of skin infections caused by *Serratia marcescens* is extremely low and such infections are typically observed in immunocompromised patients. The clinical manifestations of these infections include cellulitis, abscesses, fluctuant nodules, or granulomatous lesions. Infections caused by *S. marcescens* are very difficult to treat due to their resistance to many antibiotics, which often leads to specific and prolonged treatment. Infections after receiving a tattoo are very rare and are caused by unhygienic conditions or the inexperience of the tattooist. In this paper we present the case of a 32-year-old male with no comorbidity, who presented an abscess caused by *S. marcescens* in a area that was tattooed one month earlier. The case was resolved with surgery and antimicrobial therapy that was based on the antibiogram. To our knowledge, this is the first reported case of a *S. marcescens* skin infection following a tattoo, in the absence of immunosuppression.

## 1. Introduction

*Serratia marcescens* is Gram-negative facultative anaerobic bacillus, belonging to the* Enterobacteriaceae* family. Skin infections caused by* S. marcescens* are extremely rare and usually only occur in patients with underlying disease or who are immunocompromised.

The acute form presents as cellulitis or abscess formation. The chronic form is characterized by the appearance of fluctuant nodules or granulomatous lesions.

We present the first reported case of* S. marcescens* abscess after receiving a tattoo. A 32-year-old patient with no comorbidity presented an abscess in the cubital fossa of the left elbow. The area had been tattooed 30 days earlier. After identification of the pathogen, antibiotic therapy was administered based on the antibiogram. The appropriate antibiotic choice along with abscess drainage led to complete resolution of the lesion.

## 2. Case Report

A 32-year-old male patient presented with the only antecedent of an elbow fracture 15 years earlier, which was treated surgically. The patient got a tattoo on the fossa of his left elbow 30 days prior to his arrival at our department.

The patient arrived at the emergency department with pain, redness, and swelling of the upper left arm in the anteromedial region, accompanied by general malaise and fever. A physical examination revealed skin redness and edema in the tattooed area, with heat on palpation. Passive and active range of motion was preserved but was very limited by the pain, without distal nervous or vascular injury. He had a measurable fever of 38.5 accompanied by a general feeling of malaise. Lymphadenopathy or systemic symptoms were not evident.

In the emergency department we performed an ultrasound, which showed increased echogenicity and vascularity of the subcutaneous tissue but without evidence of fluid collection. Thus, we expanded the imaging studies with a CT scan, which revealed an alteration in the density and heterogeneity of the brachial muscle in relation to a possible myositis. Venous blood tests showed leukocytosis (18.500/mm^3^ with predominance of neutrophils -89%-) along with increased C-reactive protein (22.6 mg/dL) and erythrocyte sedimentation rate (98 mm).

**Figure 1:**
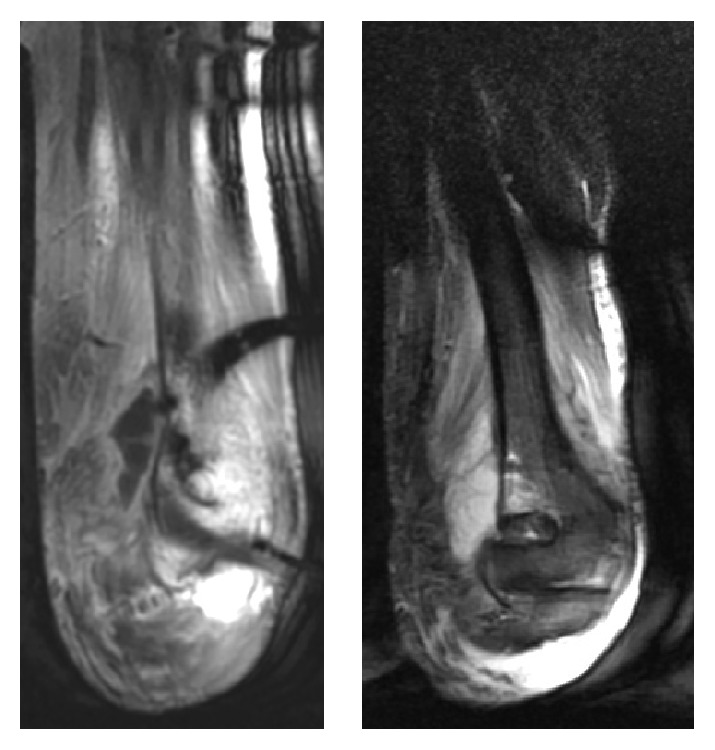
MRI (coronal T1 and STIR) showing a collection of 51 × 23 mm with bone edema in the humerus supratrochlear region with apparent cortical integrity.

**Figure 2:**
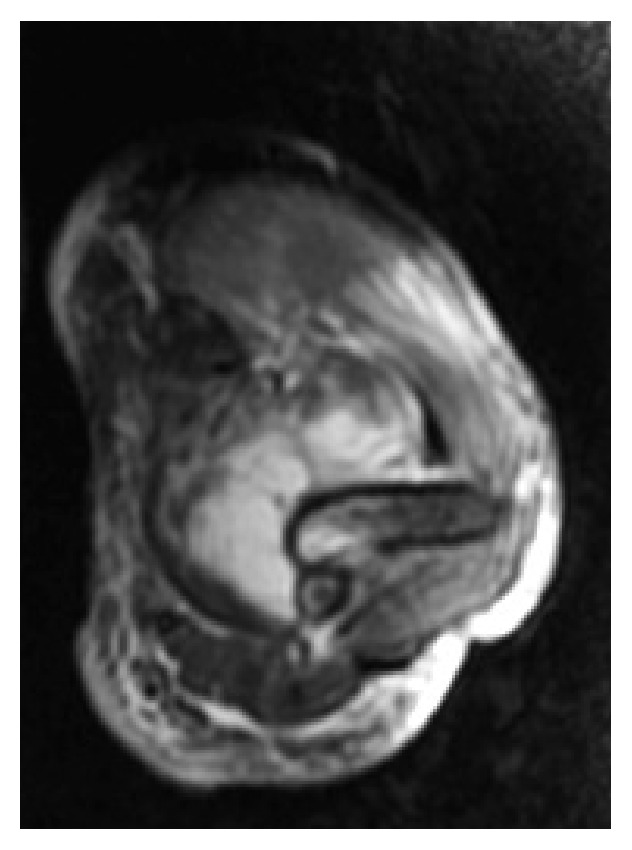
MRI STIR axial cut showing the collection.

The patient was admitted into the hospital and was treated with empirical antibiotic therapy: amoxicillin/clavulanic acid 1 g/200 mg IV + clindamycin 600 mg IV every 8 hours. To assess surgical drainage, we requested an MRI, on which a collection of 51 × 23 mm was identified. In addition, the MRI revealed bone edema in the humerus supratrochlear region with apparent cortical integrity (Figures 1 and 2).

With the additional tests results, we drained and cleaned the abscess. We used an “italic S” surgical approach to the volar aspect of the elbow and dissection by planes, finding totally unstructured tissues with abundant fibrosis. Lastly, we located and drained the abscess, which had a purulent appearance. An intraoperative sample of the purulent material was collected and cultivated and was positive for* Serratia marcescens*. During the surgery, there was an iatrogenic vascular injury in a branch of the brachial artery that required a vascular surgery suture.

In collaboration with the infectious disease unit, antimicrobial therapy was administered based on the antibiogram: ciprofloxacin 1.5 g IV with ertapenem 1 g IV daily for 3 days. On hospital discharge, oral ciprofloxacin, 500 mg every 12 hours, was prescribed for 21 days (Table 1).

Fifteen weeks after drainage, in outpatient services, the patient showed good progress with improvements in the clinical and laboratory parameters.

## 3. Discussion

*Serratia marcescens* is motile, facultative anaerobic bacillus belonging to the* Enterobacteriaceae* family. It has high survival capability under hostile conditions. It is found in nutrient-poor reservoirs such as drinking water or pipes as well as in multiple disinfectants, thus acting as a nosocomial agent. It also colonizes the gastrointestinal, respiratory, and genitourinary tract, causing opportunistic infections in addition to septicemia, arthritis, and endocarditis, which can be both nosocomial and community acquired. Skin, ocular, and soft-tissue infections are rarely reported [1, 2].

Clinical manifestations in acute skin infections are abscesses and cellulitis, which may develop into ulcers. Chronic forms are presented as nodules with an intermittent course or granulomatous lesions [2–4].

The incidence of infections with* S. marcescens* is very low, occurring in immunosuppressed patients and almost never occurring in immunocompetent patients [2, 5].

Some cases have been reported in which previous trauma, animal bites, or the presence of ulcers can serve as routes of entry for the bacteria [6, 7]. A new case has recently been published, a cutaneous facial infection after hyaluronic filler injection in an immunocompetent patient, and trauma and poor hygiene were determined to be the causes of this infection [8].

The infected area that was presented in our patient had been tattooed one month earlier. This fact, in addition to the absence of immunosuppression, suggested that the trauma of the tattoo needle injected into the skin was the gateway for the infection.

Infections after getting a tattoo are rare and are caused by inadequate hygiene and inexperience of the tattoo artist. Sometimes, the pigment used in the tattoo is infected by bacteria. Furthermore, there is an increased risk of infections when there are concomitant diseases [9].

A literature review revealed very few cases of skin infections in immunocompetent patients. The infections are predominantly observed in immunocompromised patients or in those with previously damaged skin [2, 5, 10].

To our knowledge, this is the first reported case of* S. marcescens* skin infection following a tattoo in the absence of concomitant diseases or immunosuppression. Therefore, in the case of an infection in a tattoo context, we cannot rule out* S. marcescens* as the cause.

It is important to cultivate lesion exudate for both diagnosis and correct treatment, in view of the fact that* S. marcescens* is resistant to empirical antibiotic therapy and requires specific and prolonged treatment. Consequently, in an immunocompetent patient infected with* S. marcescens*, we recommend long-term follow-up with the aim of detecting underlying immunosuppression [2].

## Figures and Tables

**Table 1:** Susceptibility results of our patient's *S. marcescens *isolate to various antibacterial drugs. The italic antibiotics were the drugs that we used in our patient's treatment.

		MIC
Amoxicillin/clavulanic acid	R	>16/8
Ampicillin	R	>16
Cefepime	S	≤1
Cefotaxime	I	2
Ceftazidime	I	8
Cefuroxime	R	>16
*Ciprofloxacin*	S	≤0.5
Cotrimoxazole	S	≤2/38
*Ertapenem*	S	≤0.5
Gentamicin	S	≤2
Piperacillin/tazobactam	S	≤8
Tobramycin	R	8

S = susceptible; R = resistant; I = intermediate.

## References

[1] Donnenberg M. S., Mandell G., Bennet J., Dolin R. (2005). Enterobacteriaceae. *Principles and Practice of Infectious Diseases*.

[2] Giráldez P., Mayo E., Pavón P., Losada A. (2011). Skin infection due to serratia marcescens in an immunocompetent patient. *Actas Dermo-Sifiliograficas*.

[3] João A. M., Serrano P. N., Cachão M. P., Bártolo E. A., Brandão F. M. (2008). Recurrent *Serratia marcescens* cutaneous infection manifesting as painful nodules and ulcers. *Journal of the American Academy of Dermatology*.

[4] Rodríguez-García F., Paz R. C., González R. S. (2006). Cutaneous infection caused by *Serratia marcescens* in a child. *Journal of the American Academy of Dermatology*.

[5] Yoshida R., Takae Y., Fujio Y., Tanaka M., Ohyama M. (2009). Cutaneous Serratia marcescens infection on the face of a healthy female. *Journal of the European Academy of Dermatology and Venereology*.

[6] Grim K. D., Doherty C., Rosen T. (2010). Serratia marcescens bullous cellulitis after iguana bites. *Journal of the American Academy of Dermatology*.

[7] Bogaert M. A., Hogan D. J., Miller E. (1991). Serratia cellulitis and secondary infection of leg ulcers by Serratia. *Journal of the American Academy of Dermatology*.

[8] Park K. Y., Seo S. J. (2013). Cutaneous *Serratia marcescens* infection in an immunocompetent patient after filler injection. *Acta Dermato-Venereologica*.

[9] Kaatz M., Elsner P., Bauer A. (2008). Body-modifying concepts and dermatologic problems: tattooing and piercing. *Clinics in Dermatology*.

[10] Langrock M.-L., Linde H.-J., Landthaler M., Karrer S. (2008). Leg ulcers and abscesses caused by *Serratia marcescens*. *European Journal of Dermatology*.

